# Prize Proposed by the Imperial Medical Society of Constantinople

**Published:** 1861-05

**Authors:** 


PRIZE PROPOSED BY THE IMPERIAL MEDICAL SOCIETY OF CONSTANTINOPLE.—This flourishing body, under whose auspices is published the *Gazette Médicale D'Orient*, offers a prize of 5000 piastres for the best essay on the medical topography of any locality or district in the Ottoman Empire. It must indicate the physico-geographical, geological, meteorological, and hygienic condition of the locality, and the causes of its insalubrity; refer to its botany and mineral waters; make a methodical examination of the popular remedies of the country; specify the various maladies, particularly epidemics; and, finally, propose the most suitable measures for curing those maladies, and for diminishing the causes of insalubrity.

